# Facile detection of peptide–protein interactions using an electrophoretic crosslinking shift assay

**DOI:** 10.1016/j.jbc.2024.107580

**Published:** 2024-07-25

**Authors:** Benjamin W. Parker, Eric L. Weiss

**Affiliations:** Department of Molecular Biosciences, Northwestern University, Evanston, IL, USA

**Keywords:** protein interaction, *in vitro* assay, methodology, peptide ligands, short linear motifs, Protein–peptide docking

## Abstract

Protein–protein interactions with high specificity and low affinity are functionally important but are not comprehensively understood because they are difficult to identify. Particularly intriguing are the dynamic and specific interactions between folded protein domains and short unstructured peptides known as short linear motifs. Such domain–motif interactions (DMIs) are often difficult to identify and study because affinities are modest to weak. Here we describe “electrophoretic crosslinking shift assay” (ECSA), a simple *in vitro* approach that detects transient, low affinity interactions by covalently crosslinking a prey protein and a fluorescently labeled bait. We demonstrate this technique on the well characterized DMI between MAP kinases and unstructured D-motif peptide ligands. We show that ECSA detects sequence-specific micromolar interactions using less than a microgram of input prey protein per reaction, making it ideal for verifying candidate low-affinity DMIs of components that purify with low yield. We propose ECSA as an intermediate step in SLiM characterization that bridges the gap between high throughput techniques such as phage display and more resource-intensive biophysical and structural analysis.

Short linear motifs (SLiMs) (1) are unstructured peptide motifs that interact with specific binding sites on folded protein domains, frequently with low to moderate affinity (1–300 μM). Such domain–motif interactions (DMIs) are a type of dynamic protein–protein association important in cellular processes such as signal transduction and protein complex assembly through multivalent contacts that can be exploited by cellular pathogens (2). The low affinity of DMIs often makes them more difficult to detect and validate than higher affinity interactions between folded proteins, thereby potentially resulting in underrepresentation in current protein–protein interaction (PPI) networks.

Straightforward pulldown or surface absorption assays are unreliable for validation of low affinity DMIs because PPIs are lost during wash steps due to nonequilibrium binding conditions (3). Instead, characterization of low-affinity interactions between peptide ligands and folded domains generally involves relatively more laborious biophysical analyses, such as fluorescence polarization (FP), isothermal calorimetry, or microscale thermophoresis, to detect and study these interactions, which also often require expensive reagents and specialized equipment. Thus, there is a need for rapid and technically simple approaches for identification and analysis of DMIs with micromolar affinity.

Here, we present electrophoretic crosslinking shift assay (ECSA), a simple and sensitive technique for detecting weak and transient PPIs, in particular DMIs, using purified component proteins. ECSA works by detecting the in-gel shift of a GFP-tagged bait SLiM peptide ligand following *in vitro* chemical crosslinking to a purified folded prey domain (Fig. 1*A*). A commonly available imidoester-based crosslinker is used to convert weak DMIs into covalent bonds. As ECSA uses two purified protein components expressed in cells, peptide synthesis is unnecessary. This technique can qualitatively detect DMIs with affinities >1 μM using less than 1 μg of purified prey protein per reaction. It can be completed in less than a day and requires only standard molecular biology reagents and commonly available gel imaging systems.

**Figure 1:**
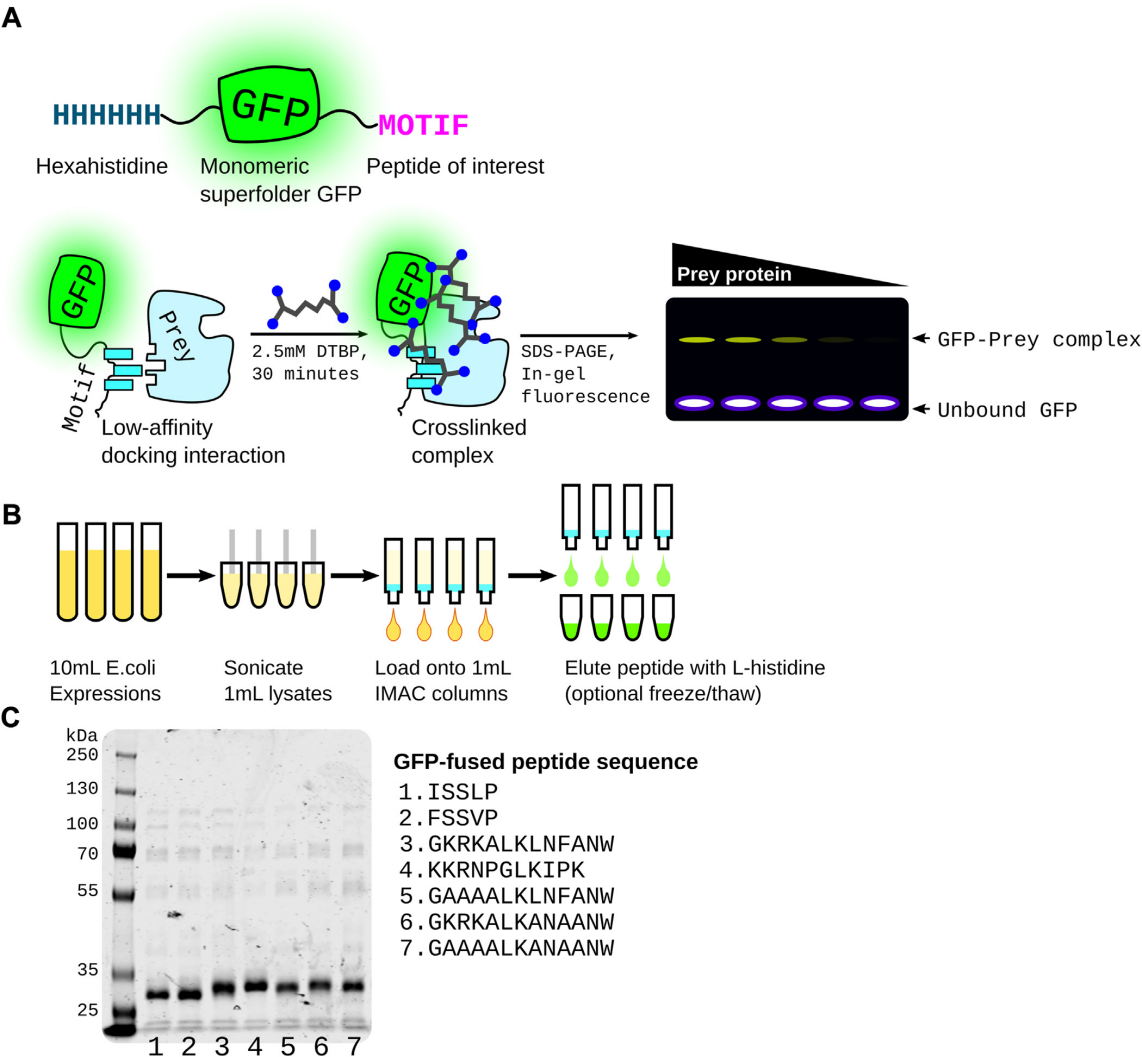
**ECSA design and workflow**. *A*, basic design of msGFP–peptide fusions used as fluorescent bait. The motif of interest is fused to monomeric superfolder GFP (7) with an N-terminal hexahistidine tag. Incubation of this motif with a prey protein in the presence of an imidoester crosslinker such as DTBP creates a crosslinked complex which can be detected by an in-gel fluorescence shift. *B*, workflow for purification of GFP–motif fusions in parallel. *C*, example of seven peptides fused to GFP as in A purified and run on Coomassie-stained SDS-PAGE. Expected molecular weight is the range of 28 to 30 kDa. DTBP, dimethyl 3,3′-dithiobispropionimidate; ECSA, electrophoretic crosslinking shift assay; msGFP, monomeric superfolder green fluorescent protein.

We demonstrate the utility of ECSA using well characterized, functionally important peptide –folded domain interactions: association of JNK1 to the MKK4 D-motif and p38α with the MKK6 D-motif, which have previously determined Kd values of ∼3.5 μM and ∼7.5 μM, respectively (4). We provide a protocol for semi-high-throughput purification of the GFP-fused bait peptide as well as for the crosslinking assay itself. We show that mutagenesis of basic and hydrophobic regions that have previously been shown to be important for D motif–kinase domain binding clearly reduces the GFP fusion protein gel shift, indicating that ECSA is a facile method for defining binding determinants in SLiM peptide ligands.

## Results

### Design and purification of monomeric superfolder green fluorescent protein–peptide fusions

We developed ECSA to assess interaction of peptide motifs with folded protein domains using peptide fusion to hexahistidine tagged monomeric superfolder green fluorescent protein (msGFP). We opted for C-terminal msGFP fusion because some SLIMs require a C-terminal carboxyl group for interaction with cognate folded domains (5), including protein trafficking signals that are mutated in human diseases (6). This variant of GFP is highly resistant to denaturation, tolerates fusion with disordered protein sequences, and refolds rapidly after denaturation by reagents such as guanidine hydrochloride (7). The hexahistidine tag coupled with the msGFP variant allows purification of bait fusion proteins under denaturing conditions, which is helpful for peptides that are prone to aggregation or bind nucleic acids due to high positive charge.

We used the well characterized interaction of the MAPK JNK with D-motif docking peptides from MKK4 and MKK6 to validate the ECSA approach, producing hexahistidine-tagged msGFP-fused D-motif peptides (Fig. 1*A*). To simulate conditions of semi-high-throughput experiments, we expressed and purified all msGFP–D motif fusion constructs in parallel from 10 ml cultures (Fig. 1*B*; see Experimental procedures section); Coomassie-stained SDS-PAGE analysis of representative purity is shown in Figure 1*C*.

### ECSA confirms MKK4-JNK1 DMI

**Figure 2:**
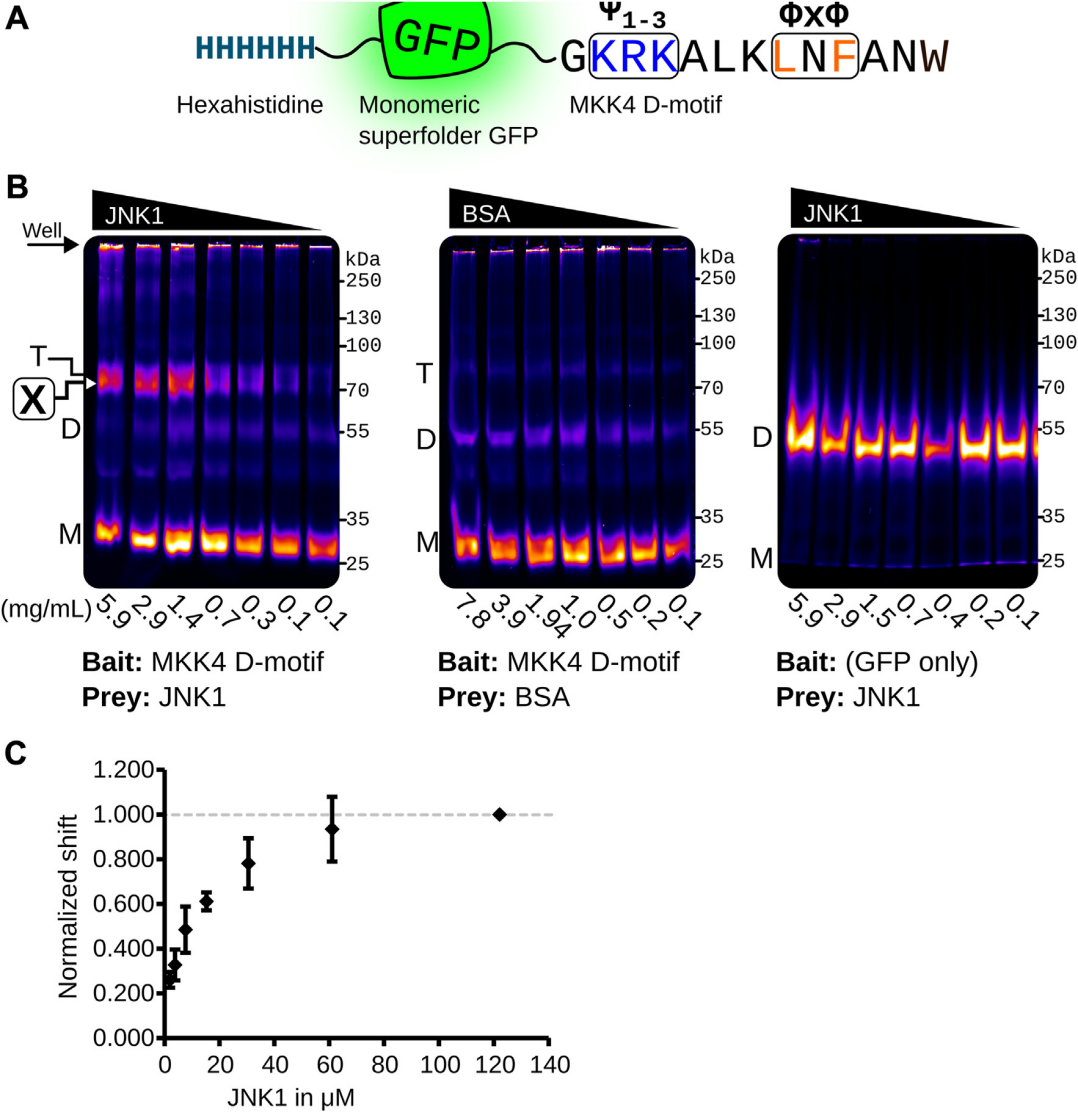
**ECSA confirms the well-characterized interaction between the MKK4 D-motif SLiM and the MAP kinase JNK1**. *A*, msGFP–MKK4 construct used as the bait peptide. The canonical basic (Ψ1-3) and hydrophobic (Φ x Φ) regions are *boxed* and highlighted as *blue* and *orange*, respectively. *B*, ECSA gel shift demonstrating binding curve with decreasing concentrations of purified JNK1 or BSA (control) as prey protein. Gel notations: (M), msGFP monomer; (D), msGFP dimer; (T), msGFP trimer; [**X**], crosslinked band representing specific docking interaction. *C*, densitometry analysis of specific [**X**] band fluorescence relative to total lane fluorescence normalized to the highest JNK1 concentration to create a standard curve. N = 3 separate crosslinking experiments, showing mean values and standard deviation. BSA, bovine serum albumin; ECSA, electrophoretic crosslinking shift assay; msGFP, monomeric superfolder green fluorescent protein; SLiM, short linear motif.

We first performed experiments with msGFP C terminally fused with the MKK4 D-motif, which like other “classical” D-motifs contains a basic region at its N terminus and a hydrophobic (Φ x Φ) pair of residues at its C terminus (Fig. 2*A*). We used ECSA to evaluate this msGFP–MKK4 fusion protein with titrated JNK1, which is known to interact directly with the MKK4 D-motif. We used msGFP as a negative bait protein, and bovine serum albumin (BSA) as a negative control prey protein. This treatment produced a shifted band at the expected molecular weight of ∼80 kDa, the combined mass of the msGFP–MKK4 fusion bait construct and the JNK1 prey (Fig. 2*B*, left panel). Crucially, intensity of this band correlated directly with increasing concentration of added JNK1. We quantified the percent shifted in each lane using the ∼70 kDa marker as a percent of the total fluorescence in that lane to determine a rough binding curve (Fig. 2*C*). Since the maximum amount of covalently linked msGFP–protein complex is dependent on a variety of factors such as the presence of proximal lysines as well as binding affinity, we normalized the curve to the maximum bait protein concentration—in this case, JNK1. Similar amounts of BSA or GFP alone did not produce a band shifted species with a binding curve (Fig. 2*B*, center and right panels).

We observed a fluorescent band at ∼55 kDa in all experiments following crosslinking that was independent of prey protein presence or concentration. This occurred dramatically in the msGFP-only experiment (Fig. 2*B*, right panel), in which the msGFP protein carries no peptide fusion, and very modestly with msGFP bait constructs with C-terminal peptide fusion regions. This is consistent with crosslinked dimers of the ∼28 kDa constructs produced by oligomerization of the msGFP domains, which is present in the assay at relatively high concentration. Evidence for dimerization of msGFP has thus far only been detected here by the ECSA approach. While our experiments suggest that this effect is dramatically reduced with msGFP constructs carrying C-terminal peptide fusions, researchers using this approach should be mindful of potential msGFP construct dimerization producing apparently shifted species, which would occur independent of the presence of prey proteins.

### Analysis of ECSA specificity using mutagenesis of MKK4 D-motif regions

**Figure 3:**
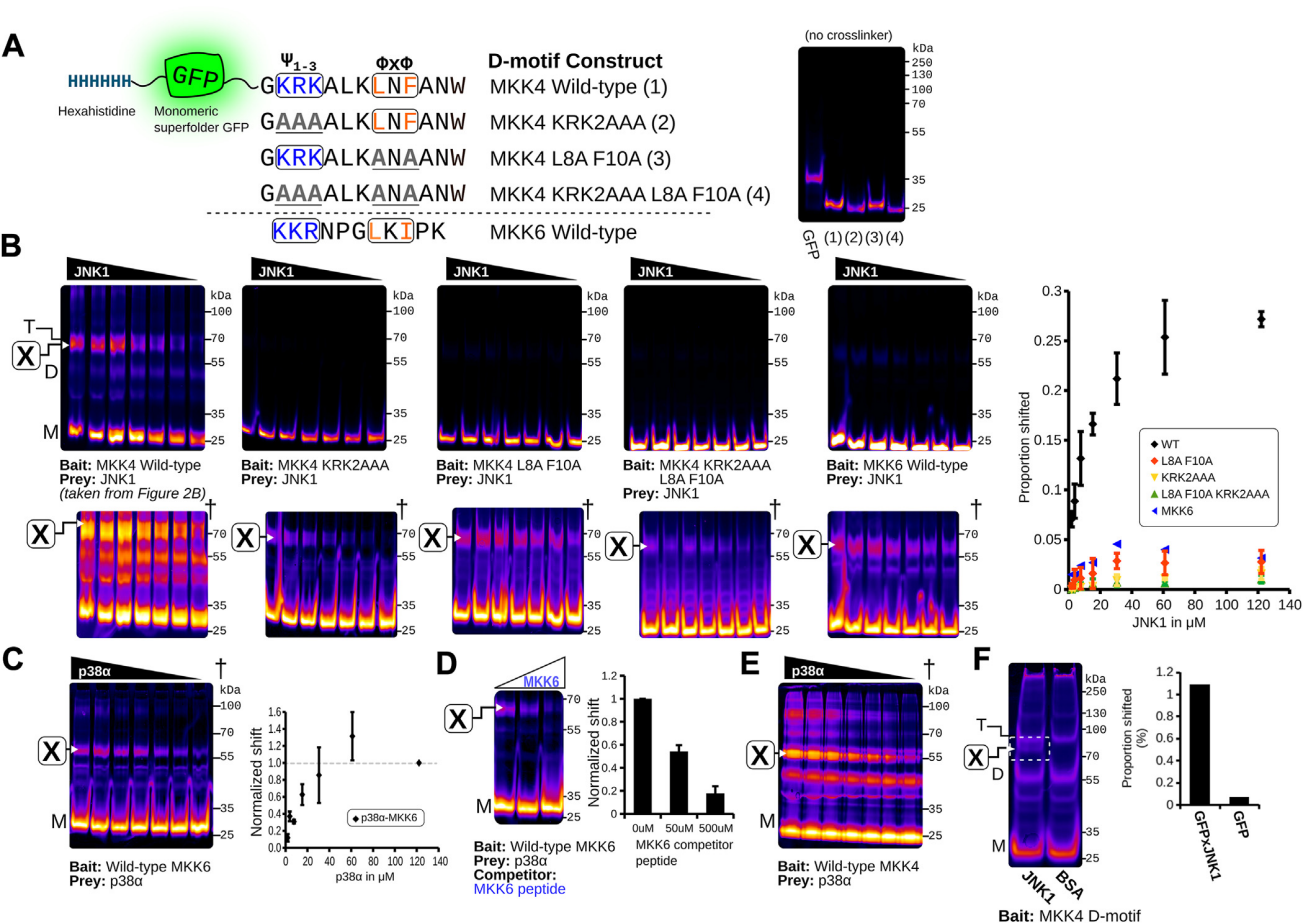
**ECSA allows assessment of multiple mutants and different D-motifs.***A*, variants of the MKK4 D-motif used to verify biochemical specificity of the assay. MKK4 peptides are labeled as (1, 2, 3, 4) for clarity. A fluorescence gel of GFP alone along with MKK4 with crosslinker omitted is shown on the *right* to show differences in migration speed between the constructs. *B*, comparative ECSA of wildtype and mutant MKK4 D-motifs (WT, n = 3; mutants, n = 2). Data from Figure 2*B* (JNK1 with WT MKK4 D-motif) is shown in the leftmost panel for comparison. Quantified shift is overlaid as a graph to the *right* and normalized to the values for the WT MKK4 D-motif. *C*, ECSA of the MKK6 D-motif incubated with p38α (n = 2). Shift is normalized to the maximum p38α concentration to show binding curve. *D*, the MKK6–p38α crosslink with increasing concentrations of MKK6 D-motif synthetic peptide as a competitor, marked in *blue* (n = 3). *E*, ECSA of p38α crosslinked to MKK4, recapitulating the published interaction. *F*, ECSA using ∼800 ng of JNK1 to demonstrate sensitivity, with quantified shifted species indicated by dashed box. Contrast adjusted (histogram equalized) images are shown with a [**X**] mark indicating the quantified bands. Binding curves and histogram with error bars represent average of three replicates with standard deviation, while histogram in *F* represents quantification of a single experiment. Images denote with dagger (†) are histogram normalized to enhance visibility of otherwise faint bands; data quantification was performed using the raw image. ECSA, electrophoretic crosslinking shift assay.

To assess the ECSA method's specificity and utility for fine structure analysis of interactions between peptide motifs and folded domains, we determined if JNK1–msGFP–MKK4 D-motif crosslinking is significantly reduced by substituting amino acids in the MKK4 D-motif known to be crucial for JNK1 interaction. Specifically, we mutated MKK4 D-motif basic and hydrophobic sequences, constructing msGFP fusion proteins bearing MKK4 D-motif mutants as shown by Figure 3*A* (basic region, KRK2AAA; hydrophobic region, L8A F10A; both regions, KRK2AAA L8A F10A). As expected, ECSA assays with JNK1 and these mutant constructs exhibited markedly reduced production of the ∼80 kDa band-shifted species compared to the wildtype, indicating a substantial loss of binding between the mutant MKK4 D-motifs and JNK1 (Fig. 3*B*).

With enhanced contrast, a faint band corresponding to the JNK1-dependent ∼80 kDa band was still observable, suggesting that binding was not entirely abolished (Fig. 3*B*; marked †). Quantification of the abundance of this ∼80 kDa band-shifted species revealed apparent binding curves for JNK1 interaction with msGFP-MKK4 D-motif mutants KRK2AAA and L8A F10A that were considerably weaker than those observed with wildtype msGFP-MKK4. Notably, these two mutant constructs each displayed more significant JNK1 crosslinking than the KRK2AAA L8A F10A mutant construct, which eliminates both conserved basic and hydrophobic portions of the MKK4 D-motif. Our ECSA mutagenesis data are largely consistent with previous studies that used pulldown assays to evaluate effects of mutations in the basic and hydrophobic portions of the D motif of 35S-labeled MKK4 on binding to GST-tagged JNK1 (8, 9).

### Analysis of ECSA using the MKK6 D-motif–p38α interaction and peptide ligand competition

The MKK6 D-motif interaction with the MAPK p38α is similar to the interaction of the MKK4 D-motif with JNK1, with a somewhat lower affinity (3.5 *versus* 7.5 μM). To further evaluate the utility of ECSA on a variety of peptide–protein complexes, we tested the interaction of the MKK6 D-motif with the purified MAPK p38α. As shown in Figure 3*C*, the msGFP–MKK6 D-motif fusion protein and p38α also showed concentration-dependent production of a band-shifted species (Fig. 3*C*). Notably, the maximum amount of shifted species was considerably weaker than the MKK4–JNK1 complex despite the comparable affinities; we speculate that this could be attributable to a lower number accessible primary amines in p38α available for crosslinking in the docked complex.

To test the specificity of the shifted ECSA band observed in the MKK6–p38α interaction, we performed a competition assay using increasing concentrations of the synthetic MKK6 D-motif (Fig. 3*D*). Indeed, addition of either 50 or 500 μM MKK6 synthetic peptide reduced the intensity of the shifted band at the molecular weight consistent with the crosslinked species. Notably, all crosslinking reactions were performed in the presence of 20 mM primary amine (L-histidine) to quench excess crosslinker, suggesting the reduction in shift is not due to the synthetic MKK6 peptide.

### ECSA detects the MKK4–JNK1 DMI with submicrogram prey protein input

DMIs often occur with folded eukaryotic proteins that may not purify in high yield, especially from a prokaryotic expression system. We found that JNK1–MKK4 binding could be detected at a final concentration of 0.2 mg/ml, even for some MKK4 D-motif mutants, suggesting that ECSA can be sensitive enough to allow use of small amounts of input bait protein (Fig. 3*F*).

## Discussion

ECSA is a fast, sensitive, inexpensive, and semi-high-throughput method for identifying micromolar affinity DMIs using *in vitro* purified components. This technique does not replace more quantitative biophysical techniques such as isothermal calorimetry or FP but is rather meant to precede them to screen many DMI “hits” that merit further study. ECSA should be a useful bridge between discovery-scale experiments like phage display and more painstaking biophysical and structural characterization. The approach should prove particularly useful when identifying sites of interaction between large, disordered proteins and folded domains because it allows “scanning” of the disordered protein-binding partner through rapid parallel query of many short motifs.

The peptide ligand that mediates MKK4–JNK1 interaction was first analyzed through laborious GST pulldowns with 35S-labeled MKK4 constructs (8, 9), a key initial characterization of MAPK docking (10). We used D-motif–MAPK interaction to validate ECSA. A few similar *in vitro* techniques exist for the discovery of weak DMIs or other PPIs. These include PUP-IT and PUP-IT2 (11, 12), which employ a bacterial enzyme, PafA, to phosphorylate a glutamate on the target peptide (Pup) fused to the bait protein. This phosphoglutamate rapidly reacts with lysine side chains on the prey to form an amide crosslink. The primary application of this technique was to detect PPIs in the cellular context, and it has been demonstrated to validate multiple *in vitro* DMIs with low to modest affinities (12). While we have not directly compared these PafA-based approaches with ECSA, we consider them as complementary strategies.

While ECSA generates qualitative binding curves for interactions with the MKK4 and MKK6 D-motifs, the method should not be interpreted as providing quantitative data. It does not precisely mirror the *in vitro*-binding affinities of these DMIs as rigorously determined by techniques like FP. This discrepancy may be attributed in part to the heightened complexity of solution dynamics involving folded domains (such as JNK1 and the msGFP fusion proteins), as well as factors like the reaction of the imidoester crosslinker with the protein, protein binding and dissociation kinetics, and quenching of crosslinking by water. Notably, JNK1 interaction affinity with D-motifs has been primarily measured using synthetic peptides. It is possible that use of this peptide ligand appended to msGFP’s globular folded domain in ECSA more closely resembles the natural context of the binding interaction.

The maximal ECSA signal produced by the interaction of msGFP–MKK4 D-motif fusion with JNK1 was significantly higher than that produced by the interaction of msGFP-MKK6 D-motif with p38α, despite only a modest 2-fold difference in previously measured Kd values. Consequently, the maximum attainable level of crosslinking of msGFP–peptide motif fusions with distinct prey proteins varies. Such variability is anticipated in crosslinking reactions in which primary amines must be sterically accessible within the kinetic time course of the reaction. Despite this discrepancy in maximal signal intensity, both JNK1 and p38α D-motif interactions produced clear qualitative binding curves upon titration of the prey protein. Therefore, we recommend evaluating the concentration-dependent production of the msGFP fusion protein band-shift species by prey titration.

### Technical notes and considerations

ECSA uses an imidoester crosslinker, such as dimethyl 3,3′-dithiobispropionimidate (DTBP). Crosslinkers with similar linker lengths, such as dimethyl suberimidate, work as well. The approach does not work well with traditional NHS crosslinkers such as BS3, which convert the positive ε-amine of lysine into an uncharged and hydrophobic amide. In practice, we found that using NHS crosslinkers causes severe smearing on the gel, possibly due to denaturation and aggregation of the bait–prey protein complex aggravated by elimination of lysine side chain positive charge.

The loading buffer used in this study, containing 1M glycine and 20% glycerol, works well for these monomeric MAP kinases. However, we have found in practice that larger protein complexes may require more aggressive denaturing conditions to produce clearer shifts (data not shown). In our hands, 10% SDS is an ideal loading buffer addition for aggregation-prone or less stable proteins that may otherwise form insoluble complexes independently of the crosslinking reaction. SDS must be added at room temperature and not boiled, or the msGFP will denature irreversibly. Addition of urea appears to cause smearing of the crosslinked band, making quantification difficult. Moreover, extremely denaturing buffers such as 7M urea/2M thiourea will irreversibly denature the msGFP at room temperature, and reducing agents should not be used.

The efficiency of crosslinking in ECSA will be influenced by the proximity of solvent accessible primary amines on interacting bait–prey protein partners. Signal will suffer if surface lysines are farther away than the crosslinker can reach. We have found that in some cases ECSA signal can be amplified by adding a soluble, long-chain diamine such as 1,4-butanediol bis(3-aminopropyl) ether at a 2:1 M ratio (data not shown). This most likely generates a long-chain imidoester crosslinker *in situ* with each imidoester reacting with each of the two ends of the diamine. We found that this treatment does not enhance detection of the interactions assayed here (data not shown), but it may improve signal in other ECSA experiments, Purification conditions of msGFP fusion proteins bear consideration. We used a lysis buffer with 2M sodium chloride to minimize coelution of nucleic acids with basic D-motif peptides fused to msGFP. In separate experiments, we found that msGFP fusion constructs containing uncharged or acidic peptides can be effectively purified using denaturing conditions (8M urea or 6M guanidinium hydrochloride), but this does not eliminate nucleic acid contamination of msGFP fusions with basic peptides such as the MKK4 D-motif (data not shown). We used L-histidine to elute msGFP–motif fusion proteins from nickel resin, which is milder than imidazole or low pH, without buffer exchange for eluted msGFP–peptide fusion proteins. We recommend alternate elution conditions or buffer exchange if prey proteins or DMIs of interest are suspected to be sensitive to L-histidine.

## Experimental procedures

### Expression of GFP-motif fusion proteins

Figure 1*B* shows our general workflow, in which peptide motifs of interest are fused C terminally to hexahistidine-tagged msGFP. We obtained peptide motif coding sequence as single-stranded DNA oligonucleotides (IDT, Inc), which we annealed and ligated into a pBH4-based vector (13) C terminal to msGFP. The construct was transformed into BL21 (DE3). Ten milliliter of terrific broth was inoculated with an overnight culture of the vectors, grown at 37 °C for 2 h, then expression induced with 0.2 mM IPTG, and growth continued at 16° C overnight.

### Procedure for the purification of GFP-motif fusion proteins

We resuspended green-hued cell pellets in 0.8 ml lysis buffer (50 mM Tris pH 8.0, 2 M NaCl) containing 1 mM freshly added PMSF, lysed cells by sonication with a microtip, and spun the resulting lysate in a benchtop microcentrifuge for 15 min. We transferred each supernatant to a separate tube containing 40 μl of 50% Ni-NTA resin (Genscript), nutated for 20 min to bind GFP fusion proteins, and then transferred the resin suspension to a 1 ml spin column (G-Biosciences). We collected msGFP-bound resin by spinning at 1000rcf, discarded the flow-through, and washed the resin once with 0.5 ml lysis buffer and once with 0.5 ml wash buffer (100 mM sodium phosphate, 50 mM sodium chloride, 20 mM imidazole, pH 7.4) by spinning at 1000× rcf. After a brief spin of the resin alone, we eluted bound protein adding 20 μl 200 mM L-histidine (pH 7.8), waiting 10 min, and spinning at 1000× rcf for 2 min.

As an optional but recommended polishing step, we froze the lysate at −20 °C and thawed at room temperature to enhance precipitation of residual contaminating proteins, then spun down at max speed. Resulting purified msGFP-peptide fusion can be frozen at −20 °C in the 200 mM L-histidine elution buffer for >3 years.

### Procedure for the purification of MAP kinases

We purified hexahistidine-tagged JNK1 and p38α as previously described (13).

### Procedure for the crosslinking assay

We quantified msGFP-peptide fusion concentration by A280 and adjusted to 50 μM, diluting into 200 mM L-histidine. Hexahistidine-tagged JNK1 was exchanged into kinase buffer (20 mM Hepes pH 8.0, 150 mM sodium chloride) using Sephadex G-25 gel filtration resin (Cytiva). BSA (Sigma) was dissolved in kinase buffer to 20 mg/ml. To crosslink, we added 0.5 μl of 50 μM msGFP-peptide fusion to 3.5 μl of JNK1 or BSA. We then dissolved the crosslinker DTBP (Thermo Scientific Pierce) in kinase buffer to final concentration of 25 mM and immediately added 0.5 μl to the protein solution, to a final volume of 4.5 μl with final concentrations of DTBP and msGFP-peptide fusion at 2.8 mM and 5.6 μM, respectively. We allowed crosslinking to proceed at room temperature for 35 min and then stopped the crosslinking reaction by adding 10 μl of quenching/loading buffer (1M glycine, 20% glycerol) with gentle mixing. We loaded reactions onto a 12% tris-glycine SDS-PAGE gel and separated at 200V for 35 min, and gels were imaged using a Sapphire imager (Azure Biosystems) using the SmartScan option.

### Gel shift image processing

We processed TIFF format raw images obtained from the Sapphire platform with the ImageJ gel analysis toolset (14). To make Figs. 2 and 3, we used the ImageJ “fire” lookup table to more clearly visualize signal intensity; to produce high contrast images for figures (denoted †), we contrast enhanced the raw image using intensity histogram equalization. We used ImageJ’s gel analysis package on the original (unmodified) TIFF image to quantify binding.

## Data availability

All data described in the “Results” section are included herein; all TIFF files from the Sapphire imager are available upon request to the corresponding author. Additional data supporting suggestions regarding loading buffer and reagents that extend crosslinker length are described in a manuscript currently in preparation and are similarly available upon request.

## Conflict of interest

The authors declare no conflict of interest with the contents of this article.
